# Detection and Molecular Characterization of Canine Distemper Virus in Wildlife from Northern Italy

**DOI:** 10.3390/pathogens11121557

**Published:** 2022-12-19

**Authors:** Tiziana Trogu, Anna Castelli, Sabrina Canziani, Clara Tolini, Maya Carrera, Enrica Sozzi, Davide Lelli, Giovanni Tosi, Laura Fiorentini, Alessandra Di Donato, Gianluca Rugna, Danilo Lanci, Antonio Lavazza, Ana Moreno

**Affiliations:** 1 Istituto Zooprofilattico Sperimentale della Lombardia e dell’Emilia Romagna (IZSLER), via A. Bianchi 9, 24125 Brescia, Italy; 2 Centro Recupero Animali Selvatici (CRAS) Rimini, Via Baracchi 47, 47923 Corpolò, Italy

**Keywords:** canine distemper, Italy, wildlife, phylogenetic analysis

## Abstract

Canine distemper virus (CDV) is a fatal, highly contagious disease found in wild and domestic carnivores. Several outbreaks have occurred in wildlife in Italy in recent years. This study aims to detect CDV in wildlife following the increasing mortality of foxes (*Vulpes vulpes*) in the Emilia-Romagna region (northern Italy) observed in 2021. Sixty-seven foxes and one badger (*Meles meles*) were subjected to necropsy followed by histological examination and were analyzed with molecular techniques to detect the presence of CDV. Of the tested animals, 16% (nine foxes and one badger) were positive for CDV. Phylogenetic analysis showed two different lineages based on complete H gene sequences. The Europe/South America-1 lineage was detected in one fox from Modena, which resembled the CDV variant associated with a previous outbreak in northern Italy in 2018, while the European Wildlife lineage was detected in animals from the Rimini province. Amino acid analysis highlighted a Y549H mutation in all sequences collected, which is commonly associated with increased virulence.

## 1. Introduction

Canine distemper virus (CDV) is an enveloped, negative-sense, single-stranded RNA virus belonging to the genus *Morbillivirus* (family *Paramyxoviridae*). It is able to infect a wide range of hosts from many different species; in particular, Canidae, Felidae, and Mustelidae are the most susceptible families reported [1].

The CDV genome encodes six structural proteins: two glycoproteins, hemagglutinin (H) and fusion (F) proteins; one envelope-associated matrix (M) protein; two transcriptase-associated proteins, phosphoprotein (P) and large polymerase (L) protein; and one nucleocapsid (N) protein [2].

The hemagglutinin gene consisting of 1824 nucleotides encodes for H protein, which is directly involved in infection and in viral tropism as it binds to the signaling lymphocyte activation molecule (SLAM, also known as CD150), a membrane glycoprotein expressed on lymphocytes and dendritic cells [3], as well as the nectin-4 receptor on epithelial cells [4].

Compared to other morbilliviruses, the H protein in CDV shows the highest variability [5]. This feature allows us to distinguish at least 19 major lineages related to different geographical areas [6,7]. In Italy, three lineages are currently recognized and, similar to CDV lineages in other countries, are mainly distributed in specific geographical regions. However, in certain cases, some areas overlap.

The Europe/South America-1 lineage is particularly diffused in northern Italy [8,9,10], while European Wildlife and Arctic lineages are mostly detected in central and southern Italy [11,12,13]. Furthermore, phylogenetic analysis focused on partial sequences of the H gene found in wild carnivores in northern Italy has highlighted the presence of two different clades within the Europe/South America-1 lineage, one originating from Bavaria and the other from the Balkans [10]. During the last CDV outbreak in 2018 in the Lombardy region (northern Italy), the two clades emerged simultaneously from different geographical valleys and slowly progressed southward [14].

The occurrence of CDV outbreaks in wildlife in Italy and other parts of Europe happens in waves, and it is generally associated with increased mortality in affected populations, in particular foxes (*Vulpes vulpes*), which are the most common wild carnivore in Italy [8]. These peaks in mortality are not so evident in domestic dogs due to the common use of vaccines [15,16].

CDV’s ability to mutate could partially explain this recurring trend, which would be consistent with re-emergence and possible increased virulence associated with genomic changes. Indeed, the H protein includes residues that are the most subjected to positive evolutionary pressure, and mutations in these specific residues could be involved in both different host tropism and virulence. The evolutionary potential of the virus and its ability to infect multiple hosts allows it to endure in the territory even if the target host species declines.

Within this mechanism, mutations of residue 549, usually associated with both host changes and increased virulence, have greater impact on virus evolution. It is hypothesized that a CDV outbreak occurred in wildlife in 2008 in southern Bavaria was characterized by severe encephalitis, which originated from a strain isolated from a dog in Hungary [17] that had undergone the Y549H mutation [18]. Other cases of increased virulence associated with the Y549H mutation have been recorded in a raccoon [19], fur-bearing animals [20,21,22], Eurasian lynx, and stone marten [23].

In 2021, an increase in red fox mortality was registered in the Emilia-Romagna (E-R) region, an area adjacent to the southern border of Lombardy. Considering the impact of CDV on wild populations, this study aimed to detect it in wild carnivores found dead in this region (Emilia-Romagna). Moreover, considering the strategic importance of the H gene and the potential implications of its mutations, we planned to characterize the different CDV genotypes detected through sequencing analysis to improve our knowledge of virus evolution and to identify any potential correlation with other outbreaks that have previously occurred in Italy.

## 2. Materials and Methods

From the beginning of 2021 until early August, an unusual mortality rate was witnessed in wild carnivores in the E-R region of northern Italy. Among all carcasses provided to the Istituto Zooprofilattico Sperimentale della Lombardia e dell’Emilia Romagna (IZSLER) for routine testing to detect specific pathogens, including the rabies virus [24], Aujeszky disease virus [25], *Trichinella* spp. [26], and *Leishmania* spp. [27], 67 foxes and one badger were also tested to detect CDV as part of a regional wildlife monitoring plan. In detail, the animals came from the Forlì-Cesena and Rimini provinces, while one fox was from the Modena province. Some foxes (n = 10) died during hospitalization at a wildlife rehabilitation center in Rimini, showing neurological symptoms suggesting CDV involvement. All 68 selected animals were submitted for necropsy. Following pathological examination, tissues (brain, lungs, stomach, intestine, and bladder) were systematically collected from each carcass for histological analysis. Suitable samples were fixed in 10% buffered formalin, processed, sectioned (at 5 μm thickness), and stained with hematoxylin and eosin (HE) for histopathology analysis. When anatomo-pathological findings suggested the involvement of some infectious diseases, further virologic and bacteriological investigations were performed.

In addition, pools of the same organs mentioned above were collected during necropsy and homogenized in phosphate-buffered saline containing 1% penicillin and streptomycin and 10% glycerol, with a 1:10 dilution (*w*/*v*). After centrifugation at 3750 rpm for 15 min, 250 μL of supernatant was used for viral genome extraction using the QIAsymphonyTM SP Instrument (Qiagen, Hilden, Germany) according to the manufacturer’s instructions. Negative and positive controls were included in the extraction.

A screening PCR to detect CDV was carried out on the highly conserved nucleocapsid (N) protein gene [28]. A fragment of 287 bp was amplified by RT-PCR using the commercial Qiagen One Step RT-PCR kit (Qiagen, Hilden, Germany). PCR was performed with a total reaction volume of 25 μL that contained 5 μL of extracted RNA and 1 μL each of 20 μM CVD P1 forward (5′-ACAGGATTGCTGAGGACCTAT-3′) and CDV P2 reverse (5′-CAAGATAACCATGTACGGTGC-3′) primers [14,29]. After reverse transcription, cDNA was denatured at 95 °C for 15 min. The reaction comprised 45 cycles of a denaturation step at 94 °C for 1 min, an annealing step at 59.5 °C for 2 min, an extension step at 72 °C for 1 min, and a final extension step at 72 °C for 10 min.

Thereafter, the complete H gene was amplified in samples positive to the first RT-PCR reaction. For this purpose, four different primer pairs were used (Table 1), and RT-PCR was conducted using a one-step RT-PCR kit (Qiagen Hilden, Germany), as previously described [18]. All the PCRs were conducted under the same conditions using the same thermocycler program [14].

The PCR products were sequenced via the Sanger method and assembled in contigs using the Lasergene sequencing analysis software package (DNASTAR, Madison, WI, USA). Afterwards, the resulting sequences were compared to the GenBank database with BLAST and then aligned with ClustalW using MEGA XI software (https://www.megasoftware.net/ accessed on 8 December 2022). The phylogenetic analysis of complete H gene sequences was performed with IQ-tree software (http://www.iqtree.org/ accessed on 8 December 2022). Parameters for the maximum likelihood method, a bootstrap analysis using 1000 replicates, and the midpoint root were set. Moreover, the best-fit model TVM + F + G4 identified by ModelFinder was applied [30,31,32]. The protein sequence analysis of the H gene was performed with Lasergene software (DNASTAR, Madison, WI, USA), highlighting a variable amino acid composition.

## 3. Results

At the necropsy examination, one-third of the carcasses analyzed were in good body condition, with no external lesions referable to infectious diseases or coat alterations. It was only possible to detect mange cases, ticks (belonging to the *Pholeoixodes ricinus* species), or fleas in a few cases. In most cases, traumatic lesions, probably due to vehicular collisions, were detected.

Generally, the most frequently observed internal findings were stomach emptiness in addition to, in some cases, melaena, gastric ulcers, or thickened and hyperemic walls. The presence of catarrhal–hemorrhagic enteritis, bladder repletion, and spleen and liver congestion characterized additional anatomo-pathological findings in the abdomen. At the thoracic level, pulmonary congestion, hematic effusion, and hemopericardium were detected.

Only a few samples from the foxes did not show severe post-mortem alterations and were suitable for histological examination. In this regard, multifocal macrovascular fatty hepatocyte degeneration and intense hyperemia of the spleen were reported. In two foxes, in addition to thoracic effusion, the lungs had collapsed and bilaterally scattered lesions of a lardaceous appearance with an infiltrative character. Histological examination confirmed cases of severe pneumonia due to numerous nematode larvae and eggs in the respiratory alveoli, with adults disseminated in the blood vessels. Numerous macrophage cells with intracytoplasmic hemosiderin pigment were present.

**Figure 1:**
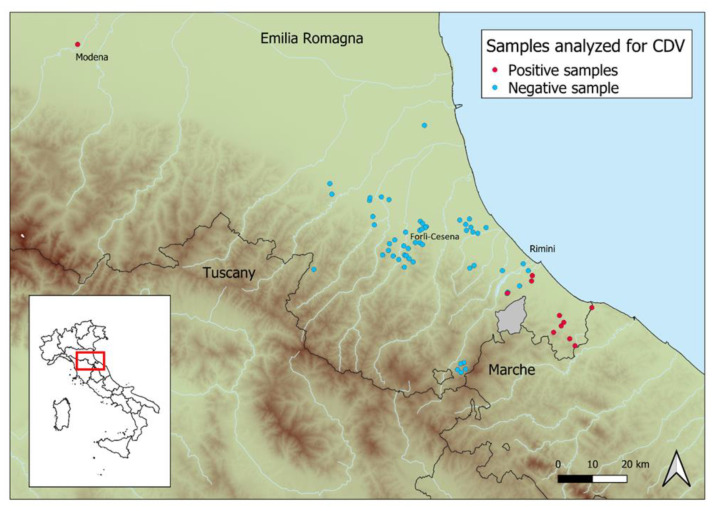
Distribution of positive (**red circles**) and negative (**blue circles**) animals.

All the samples were found to be negative after routine testing for the detection of rabies virus, *Trichinella* spp., *Leishmania* spp., and Aujeszky disease virus. In the few animals upon which bacteriological analysis was performed, *Escherichia coli* (n = 2/7) and *Pseudomonas* spp. (n = 1/7) were detected (Table S1 of Supplementary Materials). The screening RT-PCR carried out on nucleocapsid protein amplification allowed for the detection of CDV in ten foxes and one badger, showing a total prevalence of 16% (95% CI: 9–27). The positivity of nine foxes and one badger highlighted the presence of a well-defined, small outbreak in the Rimini province, near to the border with the Marche region. A single positive fox was recorded about 150 km north of the outbreak in the Modena province (Figure 1). No other pathogens were detected in the animals that tested positive for distemper.

**Figure 2:**
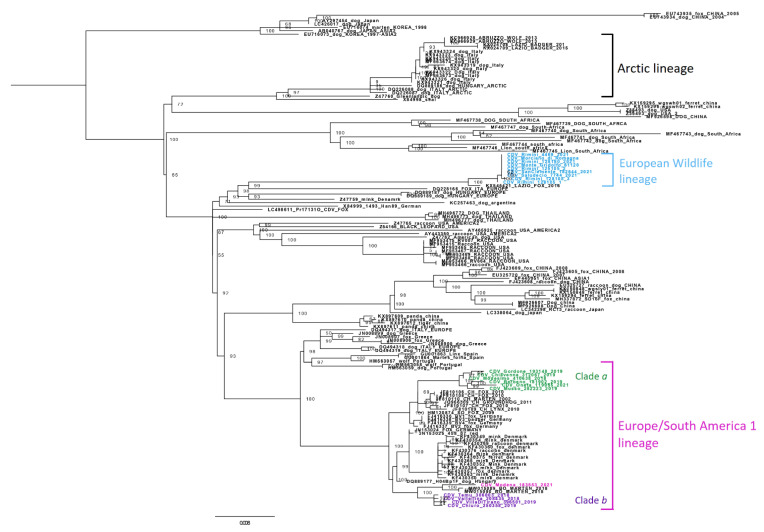
Maximum likelihood phylogenetic tree based on sequencing of the H gene. All three lineages present in Italy are shown. Italian CDV sequences investigated in this study are reported in blue (Rimini sequences in the European Wildlife lineage) and in pink (Modena sequence in the Europe/South America 1 lineage). Bootstrap values are indicated next to nodes.

Phylogenetic analysis of the complete H gene (accession numbers from OP546520 to OP546529) highlighted the detection of two different lineages: the single positive fox from the Modena province belonged to the Europe/South America-1 lineage, in particular to clade b, most likely introduced from the Balkans [10,14], and the remaining nine foxes and one badger from the Rimini province belonged to the European Wildlife lineage (Figure 2).

**Figure 3:**
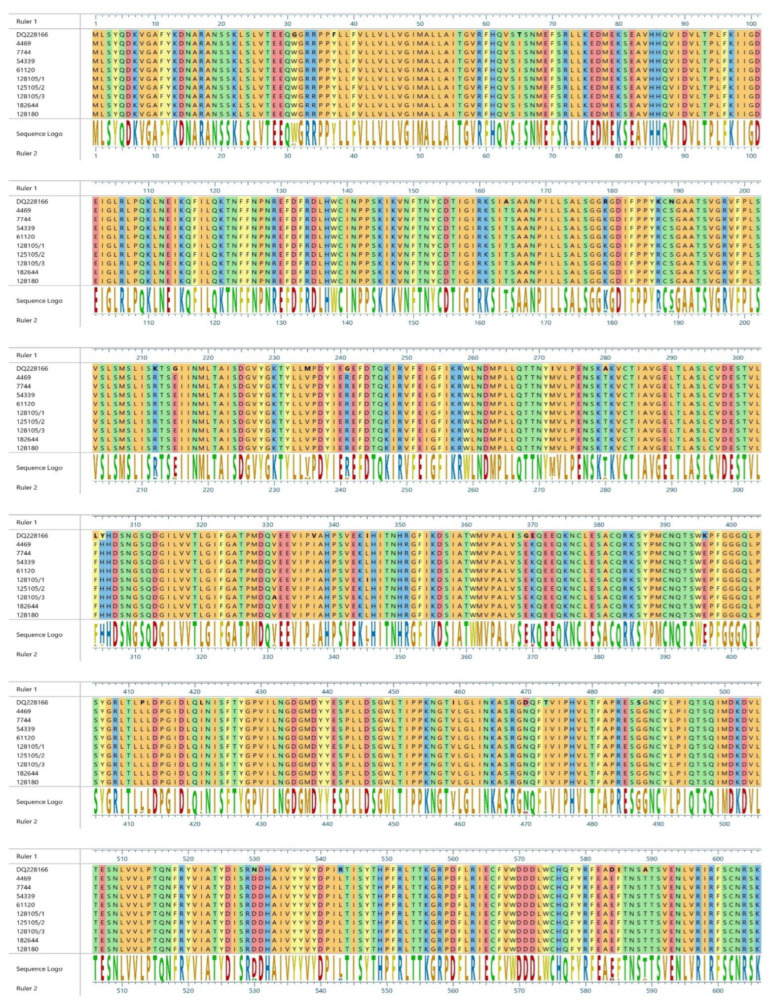
Amino acid differences among reference sequences of the European Wildlife lineage, represented by a fox collected in 2005, and sequences isolated from foxes and a badger in Rimini province.

**Figure 4:**
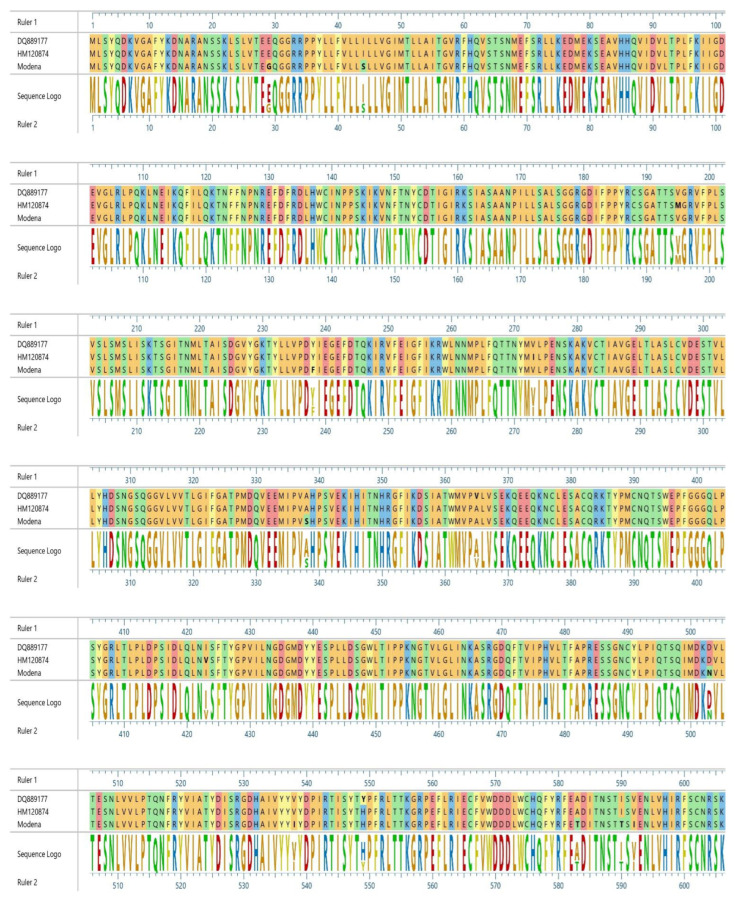
Aminoacidic differences among the Modena sequence and the reference sequences of the Europe/South America-1 lineage. In particular, DQ889177 isolated from a dog collected in 2004 represents clade *b* from the Balkans, and HM120874 isolated from a fox collected in 2009 represents clade *a* from Germany.

All analyzed sequences presented mutations from tyrosine (Y) to histidine (H) in the 549 residue. In Figure 3 and Figure 4, the main amino acid differences are shown and the reference strains of the two different lineages are compared.

## 4. Discussion

This study describes a well-defined, small outbreak of CDV in the E-R region, showing a 16% positivity rate among wild carnivores tested (in particular foxes). However, we cannot exclude the potential expansion of the infection southward, considering that most of the positive animals were found at the border of the Marche region. Unfortunately, data from these adjacent territories with different jurisdiction are currently missing.

In the E-R region, variable mortality among foxes was registered at the beginning of 2021. More than 300 foxes were conferred to IZSLER’s local laboratories across the whole region between January and August. Several studies have reported road accidents as the main cause of death for wild animals. Data on ungulate collision are surely the most abundant and registered [33], but the impact on wild populations, in particular small mammals, is well known as well [34,35]. In this regard, lesions detected in foxes reflect the predominance of traumatic injuries due to vehicle collision. Forlì-Cesena was the province that registered the highest mortality based on the number of delivered animals. Starting from the suspected circulation of CDV in foxes recovered to the wildlife rescue center in Rimini, most of the animals from the southern part of the region and other suspected foxes were analyzed to investigate its presence.

This area is characterized by the hills and mountains of the Apennines, which are particularly suitable for foxes and which could therefore lead to very high population density, increasing the likelihood of contact among potentially infected animals and therefore facilitating pathogen spread. Moreover, it is possible to highlight that the positive animals (Figure 1, red circles), except the one in Modena, came from a very limited territory, suggesting active circulation of the virus as the actual cause of death for the animals in the Rimini province. We cannot exclude the occurrence of coinfection in the foxes’ mortality; however, further analysis carried out on CDV-positive animals from January to August 2021 did not detect other viruses or bacteria of interest. The absence of other pathogens could be partially related to the poor condition of carcasses. Monitoring wildlife health status could also provide analysis on hunted animals if suitable samples were obtained. However, hunting selection could lead to biased pathological findings [36]. To include deceased animals in health screening studies is strategic, even if there are unfortunate limitations such as post-mortem alterations, difficult sample acquisition with random sampling, and progressed decomposition that could restrict both diagnostic potential and results [37].

In wildlife management, high mortality phenomena are warning bells and usually related to the emergence of an infectious disease. In this regard, several previous studies have reported cases of high mortality associated with canine distemper in relation to different carnivore species worldwide [13,14,23,38].

However, the detection of positive animals could represent the mere tip of the iceberg, and virus spread could likely be broader than what is demonstrated by genome detection analysis. First of all, CDV seems to be well adapted to wildlife. This hypothesis is supported by the cyclic emergence of CD caused by strains previously isolated and predominant in the same geographical area [39,40]. Unfortunately, in the case of subclinical manifestations or the absence of clinical forms (due to effective asymptomatic infection or lack of observation of the acute phase), the disease is likely to go unnoticed [39]. In these cases, serological investigation could help us understand pathogen spread and circulation among wild animals.

For example, a serological retrospective study carried out on badgers from Austria (2008–2020) provided identification of a high seroprevalence of CDV antibodies (43.4%) in badgers not showing neurological symptoms or other signs compatible with CD [40], supporting the hypothesis of an underestimation of this disease in wildlife. In the present study, serological data are lacking due to the impossibility of obtaining blood from conferred animals. This has certainly limited the availability of knowledge concerning the detection and level of diffusion/spread of the virus within this wild carnivore population.

Phylogenetic analysis highlighted the detection of two concomitant outbreaks in the E-R region, characterized by strains from two different lineages: sequences from Rimini’s samples were clustered within the European Wildlife lineage, while the sequence from Modena was clustered within the Europe/South America-1 lineage, and particularly with sequences grouped in clade b derived from the alpine northeastern part of Italy that originated from the Balkans.

CDV is known to be able to infect different species of animals. Residue 549 is frequently subjected to mutations that could be responsible for host range expansion and increasing virulence [8,18,23,41,42]. Indeed, in previous outbreaks characterized by severe neurological clinical manifestation [18,19,23], amino acid analysis highlighted the mutation from tyrosine (Y) to histidine (H). All the sequences recognized in the E-R region presented the Y549H mutation. Therefore, while the Modena sequence could be considered a consequence of the outbreaks that began in the Alps in 2018, characterized by a tendency to move southward, we cannot rule out the possible role of this mutations in the Rimini outbreaks, even if several factors could have influenced virus spread in this area in synergistically.

When considering the additional mutations recorded in other residues, usually not subjected to high positive selection (Figure 3 and Figure 4), it should be pointed out that, in the literature, no correlations between such mutations and changes in pathogenicity are reported. The Modena sequence showed an amino acid variation of 1.6% compared to other sequences belonging to the Europe/South America-1 lineage, while Rimini’s sequences reported an amino acid variation of 0.8% in regard to European Wildlife lineage sequences. Considering that amino acid variation between the various lineages of CDV is >4% [20], variation in the percentages recognized was very low and falls within the normal intra-lineage variation, demonstrating a close relationship between the different strains. Unfortunately, not many Italian sequences belonging to the European Wildlife lineage are available in GenBank; therefore, it is difficult to understand their evolution and epidemiology in wild populations. Moreover, several wild carnivore species populate the Apennines. Beyond foxes, badgers, and martens, wolves and bears are found along the central part of Italy. While the wolf population is steadily increasing, the Marsican brown bear (*Ursus arctos marsicanus*) is currently critically endangered. The susceptibility of these species and the circulation of different lineages in the area (European wildlife and Arctic lineages) [11,12,43] certainly complicate the epidemiological situation of the disease and open the door to several evolutionary scenarios.

In conclusion, the present study describes the contemporary occurrence of two different CDV lineages: one related to outbreaks in northern Italy in 2018–2019 caused by a strain related to the Europe/South America-1 lineage, and the other caused by the European Wildlife lineage. Even though the two groups seem to be geographically distinct, whether a southward advance of the European lineage in the future would allow for an overlap of all three lineages present in Italy cannot be ruled out. It would be interesting to continue to monitor the occurrence of CDV in wildlife and to further investigate the role of potential mutations on these populations, especially from the perspective of safeguarding endangered species.

## Figures and Tables

**Table 1:** Primers used for the amplification of the CDV complete H gene.

PCR steps	Fragments	Primer Sequence
1st PCR	72f	5′-TACTCTGGTCACACGTCTTA-3′
	798r	5′-TAGCTCCACTGCATCTGTAT-3′
2nd PCR	472f	5′-CCGTACATCACCAAGTCATA-3′
	1172r	5′-TAGAACACCACCTTGTGAAC-3′
3rd PCR	1113f	5′-GTAGATGAGAGCACCGTATT-3′
	1771r	5′-TGTGTAGGCAACACCACTAA-3′
4th PCR	1629f	5′-GGAGACCAGTTCACTGTAAT-3′
	2221r	5′-GATGGACCTCAGGATATAGA-3′

## Data Availability

Data generated or analyzed during this study are included in the published article. RNA sequencing data were deposited in GenBank, Accession Number OP546520 to OP546529.

## Supplementary Materials

The following supporting information can be downloaded at: https://www.mdpi.com/article/10.3390/pathogens11121557/s1, Table S1: Further analysis carried out on foxes.

## References

[1] Martinez-Gutierrez M., Ruiz-Saenz J. (2016). Diversity of susceptible hosts in canine distemper virus infection: A systematic review and data synthesis. BMC Vet. Res..

[2] van Regenmortel H.V.M., Fauquet C.M., Bishop D.H.L. (2000). Virus taxonomy. Seventh report of the International Committee on Taxonomy of Viruses.

[3] Tatsuo H., Yanagi Y. (2002). The morbillivirus receptor SLAM (CD150). Microbiol. Immunol..

[4] Noyce R.S., Bondre D.G., Ha M.N., Lin L.-T., Sisson G., Tsao M.-S., Richardson C.D. (2011). Tumor cell marker PVRL4 (nectin 4) is an epithelial cell receptor for measles virus. PLoS Pathog..

[5] Pomeroy L.W., Bjornstad O.N., Holmes E.C. (2008). The evolutionary and epidemiological dynamics of the paramyxoviridae. J. Mol. Evol..

[6] Panzera Y., Sarute N., Iraola G., Hernández M., Pérez R. (2015). Molecular phylogeography of canine distemper virus: Geographic origin and global spreading. Mol. Phylogenet. Evol..

[7] Wang R., Wang X., Zhai J., Zhang P., Irwin D.M., Shen X., Chen W., Shen Y. (2021). A new canine distemper virus lineage identified from red pandas in China. Transbound. Emerg. Dis..

[8] Monne I., Fusaro A., Valastro V., Citterio C., Dalla Pozza M., Obber F., Trevisiol K., Cova M., De Benedictis P., Bregoli M. (2011). A distinct CDV genotype causing a major epidemic in Alpine wildlife. Vet. Microbiol..

[9] Lorusso A., Savini G. (2014). Old diseases for new nightmares: Distemper strikes back in Italy. Vet. Ital..

[10] Bianco A., Zecchin B., Fusaro A., Schivo A., Ormelli S., Bregoli M., Citterio C.V., Obber F., Dellamaria D., Trevisiol K. (2020). Two waves of canine distemper virus showing different spatio-temporal dynamics in Alpine wildlife (2006–2018). Infect. Genet. Evol..

[11] Di Sabatino D., Lorusso A., Di Francesco C.E., Gentile L., Di Pirro V., Bellacicco A.L., Giovannini A., Di Francesco G., Marruchella G., Marsilio F. (2014). Arctic lineage-canine distemper virus as a cause of death in Apennine wolves (*Canis lupus*) in Italy. PLoS ONE.

[12] Di Sabatino D., Di Francesco G., Zaccaria G., Malatesta D., Brugnola L., Marcacci M., Portanti O., De Massis F., Savini G., Teodori L. (2016). Lethal distemper in badgers (*Meles meles*) following epidemic in dogs and wolves. Infect. Genet Evol..

[13] Balboni A., Savini F., Scagliarini A., Berti E., Naldi M., Urbani L., Fontana M.C., Carra E., Gibelli R.M.L., Gobbo F. (2021). Natural distemper infection in stone martens (*Martes foina*): From infection to neutralizing antibodies. Res. Vet. Sci..

[14] Trogu T., Canziani S., Salvato S., Bianchi A., Bertoletti I., Gibelli L.R., Alborali G.L., Barbieri I., Gaffuri A., Sala G. (2021). Canine Distemper Outbreaks in Wild Carnivores in Northern Italy. Viruses.

[15] Rikula U., Nuotio L., Sihvonen L. (2007). Vaccine coverage, herd immunity and occurrence of canine distemper from 1990–1996 in Finland. Vaccine.

[16] Martella V., Elia G., Buonavoglia C. (2008). Canine distemper virus. Vet. Clin. Small Anim..

[17] Demeter Z., Lakatos B., Palade E.A., Kozma T., Forgách P., Rusvai M. (2007). Genetic diversity of Hungarian canine distemper virus strains. Vet. Microbiol..

[18] Sekulin K., Hafner-Marx A., Kolodziejek J., Janik D., Schmidt P., Nowotny N. (2011). Emergence of canine distemper in Bavarian wildlife associated with a specific amino acid exchange in the haemagglutinin protein. Vet. J..

[19] Lednicky J.A., Dubach J., Kinsel M.J., Meehan T.P., Bocchetta M., Hungerford L.L., Sarich N.A., Witecki K.E., Braid M.D., Pedrak C. (2004). Genetically distant American canine distemper virus lineages have recently caused epizootics with somewhat different characteristics in racoons living around a large suburban zoo in the USA. Virol. J..

[20] Zhao J.J., Yan X.J., Chai X.L., Martella V., Luo G.L., Zhang H.L., Gao H., Liu Y., Bai X., Zhang L. (2010). Phylogenetic analysis of the haemagglutinin gene of canine distemper virus strains detected from breeding foxes, raccoon dogs and minks in China. Vet. Microbiol..

[21] Zhao J.J., Zhang H.L., Bai X., Martella V., Hu B., Sun Y.G., Zhu C., Zhang L., Liu H., Xu S. (2014). Emergence of canine distemper virus strains with two amino acid substitutions in the haemagglutinin protein, detected from vaccinated carnivores in North-Eastern China in 2012–2013. Vet. J..

[22] Tao R., Chen J., Zhao T., Gong C., Pan H., Akhtar R.W., Li X., Hussain Shah S.A., Li Q., Zhao J. (2020). Comparison of Growth Characteristics and Genomics of Two Canine Distemper Virus Strains Isolated From Minks in China. Front. Vet. Sci..

[23] Origgi F.C., Plattet P., Sattler U., Robert N., Casaubon J., Mavrot F., Pewsner M., Wu N., Giovannini S., Oevermann A. (2012). Emergence of canine distemper virus strains with modified molecular signature and enhanced neuronal tropism leading to high mortality in wild carnivores. Vet. Pathol..

[24] WOAH Manual of Diagnostic Tests and Vaccines for Terrestrial Animals 2022, cap. 3.1.18, par B.1.3.1.i “Rabies (Infection with Rebies Virus and Other Lyssaviruses)—Diagnostic Techniques—Identification of the Agent—Laboratory Tests—Immunochemical Identification of Rabies Virus Antigen—Fluorescent Antibody Test”. https://www.woah.org/fileadmin/Home/eng/Health_standards/tahm/3.01.18_RABIES.pdf.

[25] WOAH Terrestrial Manual 2018, cap. 3.1.2 par. B.1.2 “Aujeszky’s Disease (Infection with Aujeszky’s Disease Virus)—Diagnostic Techniques- Identification of the Agent—Identification of Virus by the Polymerase Chain Reaction”. https://www.woah.org/fileadmin/Home/eng/Health_standards/tahm/3.01.02_AUJESZKYS.pdf.

[26] Rossi P., Pozio E. (2008). Guidelines for the detection of Trichinella larvae at the slaughterhouse in a quality assurance system. Ann. Ist. Super Sanità.

[27] Galletti E., Bonilauri P., Bardasi L., Fontana M.C., Ramini M., Renzi M., Dosa G., Merialdi G. (2011). Development of a minor groove binding probe based real-time PCR for the diagnosis and quantification of Leishmania infantum in dog specimens. Res. Vet. Sci..

[28] Di Francesco C.E., Di Francesco D., Di Martino B., Speranza R., Santori D., Boari A., Marsilio F. (2012). Detection by hemi-nested reverse transcription polymerase chain reaction and genetic characterization of wild type strains of Canine distemper virus in suspected infected dogs. J. Vet. Diagn. Investig..

[29] Frisk A.L., König M., Moritz A., Baumgärtner W. (1999). Detection of canine distemper virus nucleoprotein RNA by reverse transcription-PCR using serum, whole blood, and cerebrospinal fluid from dogs with distemper. J. Clin. Microbiol..

[30] Kalyaanamoorthy S., Minh B.Q., Wong T.K.F., von Haeseler A., Jermiin L.S. (2017). ModelFinder: Fast model selection for accurate phylogenetic estimates. Nat. Methods.

[31] Nguyen L.T., Schmidt H.A., von Haeseler A., Minh B.Q. (2015). IQ-TREE: A fast and effective stochastic algorithm for estimating maximum likelihood phylogenies. Mol. Biol. Evol..

[32] Hoang D.T., Chernomor O., von Haeseler A., Minh B.Q., Vinh L.S. (2017). UFBoot2: Improving the ultrafast bootstrap approximation. Mol. Biol. Evol..

[33] Pacini M.I., Bonelli F., Briganti A., Citi S., Perrucci S., Papini R.A., Sgorbini M. (2020). Wildlife ungulate rescue and emergency services in the Pisa area (Tuscany, Italy): Evaluation of a 9-years period (2010–2018). Front. Vet. Sci..

[34] van Langevelde F., van Dooremalen C., Jaarsma C.F. (2009). Traffic mortality and the role of minor roads. J. Environ. Manag..

[35] Fusillo R., Romanucci M., Marcelli M., Massimini M., Della Salda L. (2022). Health and Mortality Monitoring in Threatened Mammals: A First Post Mortem Study of Otters (*Lutra lutra* L.) in Italy. Animals.

[36] Sleeman J.M., Brand C.J., Wright S.D. Strategies for Wildlife Disease Surveillance. Chapter 37 in Applied Techniques of Conservation Medicine 2012; USGS Staff -- Published Research. 971. http://digitalcommons.unl.edu/usgsstaffpub/971.

[37] Stallknecht D.E., Childs J.E., Mackenzie J.S., Richt J.A. (2007). Impediments to wildlife disease surveillance, research, and diagnostics. Wildlife and Emerging Zoonotic Diseases: The Biology, Circumstances and Consequences of Cross-Species Transmission.

[38] Sakai K., Nagata N., Ami Y., Seki F., Suzaki Y., Iwata-Yoshikawa N., Suzuki T., Fukushi S., Mizutani T., Yoshikawa T. (2013). Lethal canine distemper virus outbreak in cynomolgus monkeys in Japan in 2008. J. Virol..

[39] Pope J.P., Miller D.L., Riley M.C., Anis E., Wilkes R.P. (2016). Characterization of a novel canine distemper virus causing disease in wildlife. J. Vet. Diagn. Investig..

[40] Oleaga Á., Vázquez C.B., Royo L.J., Barral T.D., Bonnaire D., Armenteros J.Á., Rabanal B., Gortázar C., Balseiro A. (2022). Canine distemper virus in wildlife in south-western Europe. Transbound. Emerg. Dis..

[41] Nikolin V.M., Wibbelt G., Michler F.U.F., Wolf P., East M.L. (2012). Susceptibility of carnivore hosts to strains of canine distemper virus from distinct genetic lineages. Vet. Microbiol..

[42] Di Blasio A., Irico L., Caruso C., Miceli I., Robetto S., Peletto S., Varello K., Giorda F., Mignone W., Rubinetti F. (2019). Canine distemper virus as an emerging multihost pathogen in wild carnivores in northwest Italy. J. Wildl. Dis..

[43] Ricci I., Cersini A., Manna G., Marcario G.A., Conti R., Brocherel G., Grifoni G., Eleni C., Scicluna M.T. (2021). A canine distemper virus retrospective study conducted from 2011 to 2019 in central Italy (Latium and Tuscany Regions). Viruses.

