# Supplementary material for: Detection and Molecular Characterization of Canine Distemper Virus in Wildlife from Northern Italy
Source: Pathogens. 2022 Dec 19;11(12):1557. doi: 10.3390/pathogens11121557 (PMC9782791; doi:10.3390/pathogens11121557)
Supplement: Supplementary file 1 [file pathogens-11-01557-s001.zip › pathogens-2010084-supplementary.pdf]

Table S1. Further laboratory analysis carried out on foxes' samples. Seven carcasses only were considered suitable for performing bacteriological examination

Analysis	Num. positive (p%)
Rabies virus	0/67 (0%)
Trichinella spp.	0/67 (0%)
Leishmania spp.	0/67 (0%)
Aujeszky disease virus	0/67 (0%)
**Bacteriological analysis**	
Escherichia coli	2/7 (28%)
Pseudomonas spp.	1/7 (14%)
